# The structure of the Hallstatt evaporite body (Northern Calcareous Alps, Austria): A compressive diapir superposed by strike-slip shear?^☆^

**DOI:** 10.1016/j.jsg.2013.12.008

**Published:** 2014-03

**Authors:** Anja Schorn, Franz Neubauer

**Affiliations:** Department of Geography and Geology, University of Salzburg, Hellbrunnerstraße 34, 5020 Salzburg, Austria

**Keywords:** Diapir, Shear zone, Hallstatt, Haselgebirge, Evaporite, Vein

## Abstract

Based on old detailed mining maps and own observations in the Hallstatt salt mine, we reinterpret the structure of the Hallstatt evaporite body of the Upper Permian to Lower Triassic Haselgebirge Fm. within the Northern Calcareous Alps (NCA). The Haselgebirge Fm. represents a rocksalt mylonite with abundant lenses of sulphates, mudstones and limestones. In comparison to published results of analogue modeling we interpret the present shape of the Hallstatt body as a WNW–ESE elongated compressive teardrop-like diapir. This is overprinted by NNE–SSW shortening and dominantly sinistral shearing along a W-trending shear zone. The internal structure shows steeply dipping rock units and foliation. Earlier dextral ductile shear fabrics of likely late Early Cretaceous age are preserved in sulphate rocks and are subsequently overprinted by mylonitic fabrics in rocksalt and cataclastic fabrics in other rocks.

The low strength of halite results in recent subvertical shortening and a strain rate $\dot{\varepsilon }$ of 8 × 10^−10^ [s^−1^] is deduced from deformed subhorizontal boreholes. This value is similar to such strain rates (10^−10^ to 10^−9^ s^−1^) estimated by the halite grain size distribution from other salt mines in the NCA and thus indicative of sub-recent formation of the halite microfabrics.

## Highlights

• The present shape of the Hallstatt body is a WNW–ESE elongated compressive diapir.
• The diapir was overprinted by N–S to NNE–SSW shortening.
• The dominant sinistral shearing mainly took place along a W-trending shear zone.
• Early ductile shear zone fabrics are preserved in anhydrite and polyhalite rocks.
• Recent subvertical shortening was deduced from deformed boreholes within rocksalt.

## 1 Introduction

Within mountain belts, evaporite bodies tend to develop complex shapes because of the superposition of several sequential deformation events. Evaporites of mountain belts are usually deposited in the rift stage of passive continental margins at the transition from initial terrestrial to subsequent shallow marine conditions. This predates the plate-tectonic drift stage at ocean margins. Because of the early deposition and the low strength of halite, many evaporite layers are deformed by raft tectonics during the drift stage (e.g., Butler and Paton, 2010) and by a succession of shortening deformation stages during ocean consumption and continental plate collision. Earlier formed salt walls and diapirs are affected by compressive deformation and could become compressive rootless diapirs (highly mature diapirs by Jackson and Talbot (1989)) or salt nappes.

For compressive settings, analog experiments testify significant differences between thick evaporitic décollement levels and thin décollement horizons. Considering a thick evaporitic layer, compressive structures are symmetric and upright compressive diapirs and associated double-vergent thrusts as well as wide synclines and small anticlines develop (e.g., Warren, 2006, Hudec and Jackson, 2007 and references therein). In the case of a thin evaporite layer, foreland-directed vergent structures arise.

In this contribution, we discuss the example of the Hallstatt rocksalt body within the Northern Calcareous Alps (NCA), which represents one of the oldest active salt mines throughout the world. Well-documented mining maps and sections allow the reconstruction of major portions of the evaporite body (Schauberger, 1949, Schauberger, 1955, Schauberger, 1986 and references therein), which is exposed within a classical fold-thrust belt (Linzer et al., 1997, Linzer et al., 2002). Together with structural observations in the mine, these examples enable the reconstruction of the succession of deformation phases, which shaped this particular rocksalt body.

## 2 Geological setting of the NCA and the Hallstatt region

**Fig. 1:**
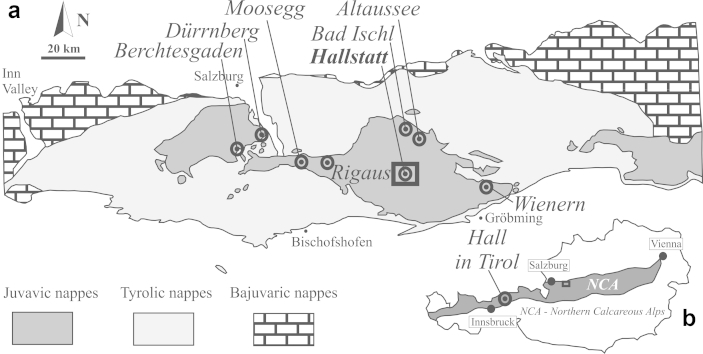
a – Overview of Austroalpine units in the central Northern Calcareous Alps (NCA) (modified from Leitner et al., 2011). b – Inset shows distribution of the NCA in the frame of Austria (modified after Schorn et al., 2013a).

The Hallstatt salt mine is located within the Upper Permian to Lower Triassic Haselgebirge Fm. of the NCA, whose formation ages range from Late Carboniferous to Eocene. The classic division within the NCA defines the Bajuvaric, Tyrolic and Juvavic nappe complexes (Tollmann, 1985, Mandl, 2000 and references therein) (Fig. 1). The lagoonal Haselgebirge Fm. occurs mainly in Juvavic units of the central and eastern NCA and subordinately in Tyrolic units (Tollmann, 1985, Schauberger, 1986, Leitner and Neubauer, 2011 and references therein). The lagoonal Haselgebirge Fm. marks the transition from mostly terrestrial Permian siliciclastic successions to shallow and deep marine carbonate-dominated sequences since the Middle Triassic. The Upper Juvavic nappes comprise mostly Middle to Upper Triassic reefs and deposits next to reefs. The Lower Juvavic nappe unit represents outer shelf or intrabasinal ridges with reduced sedimentary thickness (pelagic Hallstatt Limestone) (Tollmann, 1985, Mandl, 2000). The ridges are suggested to relate to salt diapirism in Triassic times (Mandl, 1982, Mandl, 2000, Plöchinger, 1984). The Permian/Middle Triassic- to Upper Triassic succession is interpreted as the rift stage and passive margin formation of the Hallstatt-Meliata Ocean (Lein, 1987). The Hallstatt-Meliata Ocean was closed during the Late Jurassic (Dallmeyer et al., 2008 and references therein). Evidence of gravitational sliding was reported from different places (e.g., Mandl, 1982) and this concept has been developed until recent years, arguing for a Late Jurassic age of shortening (Missoni and Gawlick, 2011).

During the Early Cretaceous, nappe stacking of Austroalpine units began with the subduction of Austroalpine continental crust and thrusting prograded from ESE to WNW (Ratschbacher, 1986, Linzer et al., 1997, Mandl, 2000, Neubauer et al., 2000).

The mechanism and the time of emplacement of the Juvavic units are still debated. The classic hypothesis assumes that both Juvavic nappes were emplaced during the eo-Alpine thrusting (late Early to early Late Cretaceous; Kober, 1955, Pichler, 1963, Schweigl and Neubauer, 1997, Mandl, 2000, Schorn and Neubauer, 2011). An alternative model explains the emplacement of all Juvavic units by gravity sliding of mountain-size blocks (olistostromes) during the Late Jurassic, as Haselgebirge clasts were found in the Upper Jurassic Oberalm and Lower Cretaceous Rossfeld formations (Missoni and Gawlick, 2011 and references therein). The Cretaceous nappe stack within the NCA was overlain by Upper Cretaceous to Eocene Gosau sediments (e.g., Wagreich and Decker, 2001), which have formed in a piggy-back or collapse basin setting.

In the Eocene, after consumption of the Penninic Ocean, the second paroxysm of the Alpine orogeny occurred, when the European continental basement was subducted below the NCA at the leading edge of the Austroalpine-Adriatic microcontinent (Faupl and Wagreich, 2000, Linzer et al., 2002). The NCA was thrust over the Rhenodanubian Flysch (part of the Penninic Ocean fill) and Helvetic domain (cover of the European continent) resulting in a wide thin-skinned tectonic nappe complex (Linzer et al., 1997, Mandl, 2000, Neubauer et al., 2000). Deformation of the Upper Cretaceous to Eocene Gosau basin fill deposited on uppermost nappes (Tyrolic and Juvavic nappes) suggests significant post-Gosau deformation in Late Eocene to Early Miocene times (Linzer et al., 1997, Peresson and Decker, 1997).

Salt mining has a long tradition in the central NCA and has started in Hallstatt ca. 3500 years ago (Kern et al., 2008). In the Bronze Age and Early Iron Age the settlement developed to a flourishing mining centre culminating in the “Hallstatt culture” (European Early Iron Age, ∼ 800–400 BC).

**Fig. 2:**
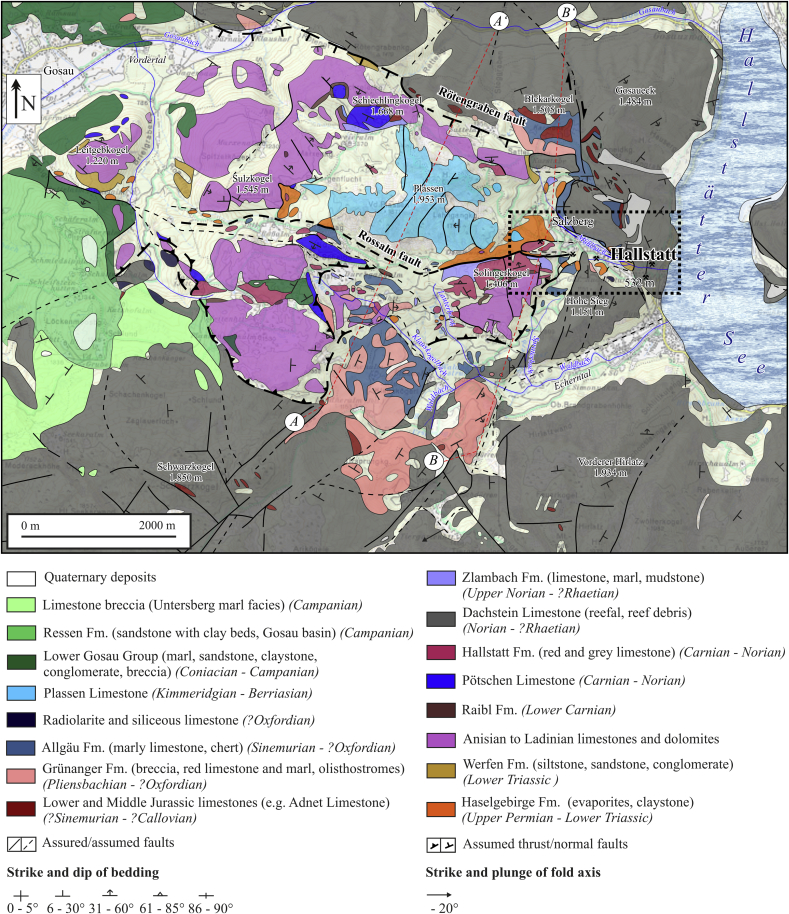
Simplified geological map of the Hallstatt region (modified after Plöchinger, 1982, Schäffer, 1982, Mandl, 1998). Aʹ – A and Bʹ – B mark the approximate locations of cross-sections A and B in Fig. 3. The rectangle exhibits the approximate location of surface facilities of the mining area.

**Fig. 3:**
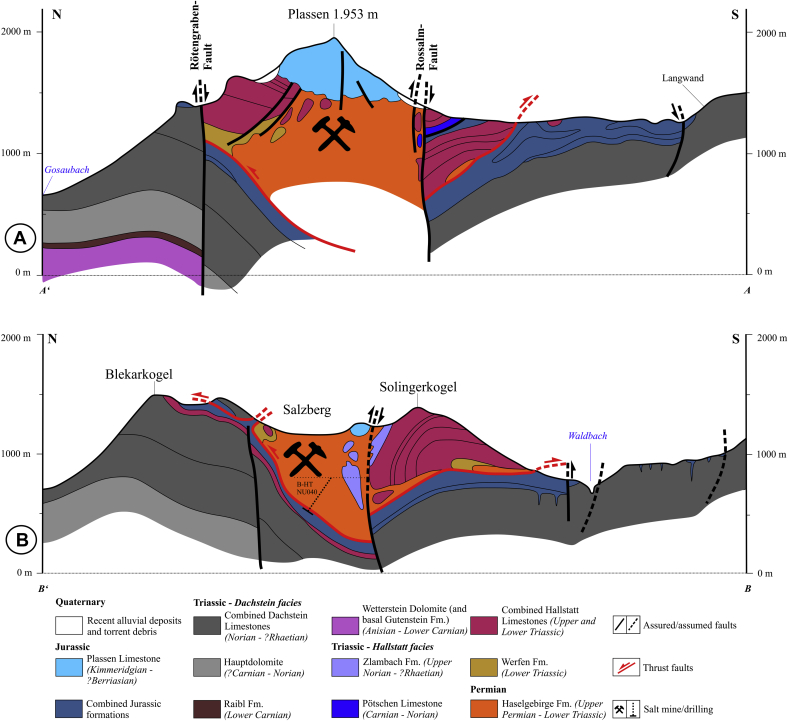
Geological cross-sections through the Hallstatt region (modified after Mandl et al., 2012). For approximate locations of cross-sections A and B see Aʹ – A and Bʹ – B in Fig. 2.

The salt body of Hallstatt (Figs. 2 and 3) lately considered as Hallstatt nappe (Habermüller, 2005), was recently described by Leitner and Neubauer (2011) and here we follow their description. The Haselgebirge body extends ca. 3 km in E–W-direction and is around 600 m wide (maps and sections: Schauberger, 1955, Schäffer, 1982, Scheidleder et al., 2001, Habermüller, 2005). The halite content of the Haselgebirge Fm. is around 55 weight % (Schauberger, 1949, Schauberger, 1955) but varies between lithologies. The borders and the salt layers dip ca. 40° to the North and steepen to 70° at depth. The salt mine extends vertically from 514 m to 1267 m above sea level, whereas the salt body suitable for mining diminishes with depth, mostly because of inclusions of country rocks. The base of the salt was not reached in workings down to 100 m below the level of Lake Hallstatt (pers. comm., Gerald Daxner, Salinen Austria AG).

The continuation of the Haselgebirge body toward Mount Plassen (located above the western part of the salt body; Figs. 2 and 3) is poorly known (i.a. Arnberger, 2006). According to Mandl et al. (2012, Fig. 3) in combination with older detailed profiles of Schauberger (unpublished data of the Salinen Austria AG) the salt body of Hallstatt can be seen as a WNW–ESE-elongated compressive diapir.

The sedimentary succession of the Lower Juvavic unit is nearly complete and comprises, besides the basal Haselgebirge Fm., Lower Triassic to Liassic formations. The Haselgebirge Fm. also contains rare blocks of meta-basalts (melaphyre; Zirkl, 1957) within the colored salt-bearing claystone (“Buntes Salztongebirge”; Schauberger, 1986).

As was revealed by several studies using pollen and sulfur isotopes (Klaus, 1965, Spötl, 1988a, Spötl, 1989, Spötl and Pak, 1996), the sediments of the Haselgebirge Fm. were deposited during Late Permian and Early Triassic times. During the Late Permian, the Haselgebirge Fm. was deposited under subtidal to supradital marine conditions (Spötl, 1988b), whereas the Lower Triassic is dominated by the shallow marine and intertidal sedimentation of clastic and carbonate-clastic rocks (Werfen Fm.).

Permian clastic sediments (Spötl, 1987) and Hallstatt Limestone (Krystyn, 2008) are often associated with Jurassic rocks (Sandlingalm Fm.). Blocks of the Hallstatt Limestone are in contact with rocks of the Sandlingalm Fm., which, together with the Haselgebirge Fm., are covered by a lid of undeformed Upper Jurassic rocks (Gawlick and Schlagintweit, 2006, Suzuki and Gawlick, 2009). A sedimentary contact between the Sandlingalm Fm. and the overlying Plassenkalk Fm. is missing (Suzuki and Gawlick, 2009) and the Lower Juvavic unit, the rocksalt and the Jurassic cover are surrounded by the Upper Juvavic nappe (Fig. 2).

Based on vitrinite reflectance studies, maximum temperatures for the central sectors of the NCA in the surroundings of the Hallstatt mine are estimated between 160 and 180 °C (Spötl and Hasenhüttl, 1998, Leitner et al., 2011).

## 3 Structure of the Hallstatt body

The Hallstatt salt body is exposed at the eastern termination of a large W-trending zone of Haselgebirge Fm. (Fig. 2). Based on drilling data provided by the Salinen Austria AG for the Salzkammergut area, profiles of Mandl et al. (2012; Fig. 3) show a steeply south-dipping salt body, which is “squeezed” between thick Triassic and Jurassic sequences. Thrust faults occur along the northern margin and steep faults along the southern margin. There are also splays with S-directed thrust faults (Fig. 3). In general, the northern margin dips steeply S, the southern margin is the W to WNW-trending Rossalm fault on the maps (Figs. 2 and 3), which is subvertical and includes both features of a reverse fault with a northern block up-kinematics and a strike-slip fault.

**Fig. 4:**
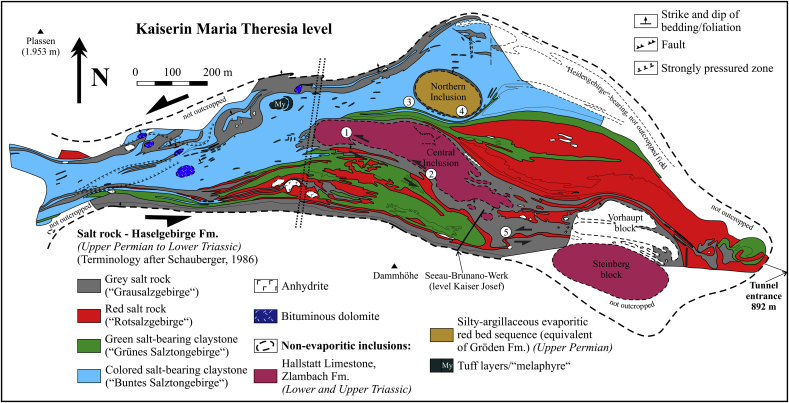
Map view of Kaiserin Maria Theresia level of Hallstatt salt mine at an altitude of 892 m above sea level (tunnel entrance) showing various shear structures (modified after Schauberger, 1955). Labels 1–5 denote specific structures described in the text. The western part approximately to the left of the stippled double lines is the result of overall sinistral shear. The eastern part is dominated by overall NNE–SSW shortening.

**Fig. 5:**
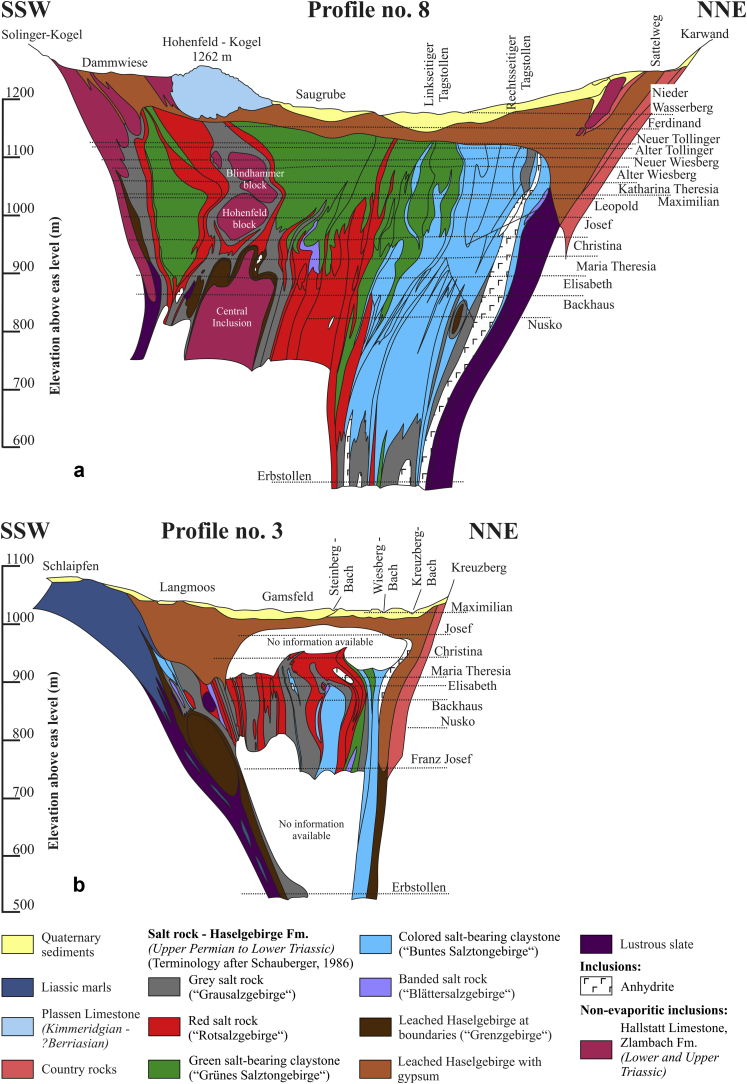
Two detailed ca. SSW–NNE-trending geological sections across the Hallstatt salt mine (cross-sections 8 and 3 of Othmar Schauberger, Salinen Austria AG), which clearly show that the salt body is smaller at depth and widens up-section. a – Central part of the salt deposit. b – Eastern part of the salt deposit.

**Fig. 6:**
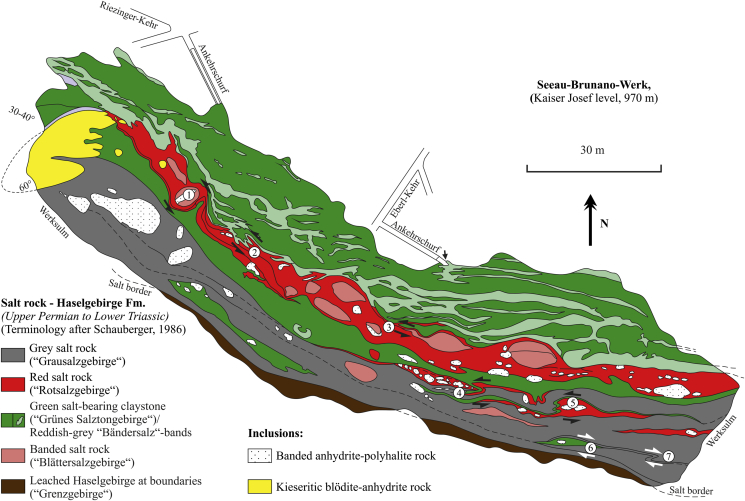
Detailed map view of the roof of the solution cavern “Seeau-Brunano-Werk” at the southern border of the salt body (modified after Schauberger, 1949). Labels 1–7 denote structures described in the text. Note the sinistral shear sense indicators along the main red rocksalt body and dextral shear along the southern margin. For approximate location of the solution cavern, see Fig. 4.

The Hallstatt salt mine comprises 21 levels and several smaller shafts ranging from 514 m above sea level (Erbstollen level) to an elevation of around 1267 m (Erzherzog Matthias Schurf level). In the past, Schauberger, 1949, Schauberger, 1955, Schauberger, 1986 and references therein) mapped the mine in great detail (e.g. Fig. 4) and drew a series of N–S and NNE–SSW-cross-sections, of which we only show two significant profiles (Fig. 5) together with a map view from one solution cavern (Fig. 6).

We follow the rock type definitions for the Haselgebirge Fm. of Schauberger (1986). Greenish and reddish rocksalt is often mixed with significant portions of mudstone (20–80%), subordinate anhydrite or polyhalite rocks (Schorn et al., 2013a), rare meta-diabase (Zirkl, 1957) as well as large inclusions of country rocks and many centimeter- to hundred-meter scaled rock fragments (limestones, marls and sandstones). The largest of these incorporated lenses are often elongated about parallel to the main strike direction (Schauberger, 1949, Schauberger, 1955) and are referred to by the salt-miners as the “Central Inclusion” (“Zentrale Einlagerung”), the “Vorhaupt block” (“Vorhaupt Scholle”) and the “Northern Inclusion” (“Nördliche Einlagerung”) (Figs. 3 and 4 and Fig. 5a). The about 200 m wide and more than 1000 m long “Central Inclusion” (Fig. 4 and Fig. 5a) forms the core of this structure (Schauberger, 1949, Arnberger, 2006). It consists of Hallstatt Limestone and Zlambach Fm. and is dissected in its upper parts into smaller lenses, whereas the easternmost one is called the “Steinberg block” (“Steinberg Scholle”) (Fig. 4). The maximum ca. 200 m wide “Northern Inclusion” represents an Upper Permian silty-argillaceous evaporitic red bed sequence (Spötl, 1987) with frequent carbonate concretions, intersected by graded sandstone intercalations with Upper Permian clay-sherds and plant remains (Spötl, 1987) as well as spores and pollen (Klaus, 1987). The “Northern Inclusion” was most likely deposited in a marginal marine shallow water environment with gradually increasing salinity (Spötl, 1987).

The internal structure and the largest external extent of the Hallstatt salt mine is shown in Kaiserin Maria Theresia level (Fig. 4). At this level, a ca. rectangular WNW-trending body in the East with thick lithologies narrows towards West forming a small “tail”.

We describe – based on the mining maps and sections (Fig. 4, Fig. 5, Fig. 6) – the kinematically relevant structural features:

As mentioned above, nearly the whole Hallstatt salt body strikes E–W to ESE–WNW. Towards greater depth, the wide upper parts narrow and most lithologies can be traced although they thin out (Fig. 5). The boundaries of the salt body consist of dark, mainly salt-free mudstones and lustrous slate (“Glanzschiefer”) showing abundant slickensides. Inclusions, which represent rigid bodies of country rocks, are often aligned parallel to the boundaries and are more fragmented towards the top. The evaporite body is capped by leached Haselgebirge, which is ca. 50–100 m thick (Fig. 5).

Only a steep Haselgebirge lens exposed along a 150 m long outcrop of the Erbstollen level within Norian Dachstein Limestone (directly juxtaposing the Permian Haselgebirge Fm.), strikes NNE–SSW. Its foliation is orientated about perpendicularly to the strike of the main part of the salt body and dips to the West (Schauberger and Medwenitsch, 1951, Arnberger, 2006). According to Arnberger (2006), this indicates that the Haselgebirge evaporites might have acted as a detachment horizon for W-directed thrusting before their migration into the strike-slip fault system.

We divided the Kaiserin Maria Theresia level (Fig. 4; Schauberger, 1955) into two parts with distinct fabrics: The eastern part is dominated by inclusions with a low aspect ratio (length/width in map view) elongated mostly in WNW–ESE-direction. These observations imply overall shortening in ca. NNE–SSW- to NE–SW-direction. In the western part (approximately to the left of the stippled double lines in Fig. 4) all the Haselgebirge zones are heavily stretched and thinned in E–W-direction. This indicates thinning as the result of overall sinistral shear. Therefore, this event postdates NNE–SSW shortening as, e.g., the “Central Inclusion” is dragged into the sinistral shear zone. In the map view, many shear sense indicators can be observed. These include asymmetric shear folds (labels 1, 2 and 5 in Fig. 4 and labels 2, 4 and 5 in Fig. 6), delta-like fringed clasts (label 1 in Fig. 6) and sigma-like shear bands (labels 3, 6 and 7 in Fig. 6). Large isoclinal folds with a fold closure in the West are present in green salt-bearing claystone (e.g., label 3 in Fig. 4). Nearly all map-scale shear sense indicators exhibit sinistral shear except along the margins (labels 6 and 7 in Fig. 6). The stiff inclusions affect the trace of foliation within neighboring rocksalt and claystone and the weak lithologies wrapped around these inclusions (label 4 in Fig. 4). We note, therefore, reasonable evidence for sinistral shear along an about W- to WNW-trending zone and less important dextral shear along the southern margin.

## 4 Evaporite fabrics and deformation history

In the following, we describe some significant structural arrangements from the interior of the evaporite body. All the structural orientation data is plotted using the program TectonicsFP (Ortner et al., 2002).

### 4.1 Structural assemblages

**Fig. 7:**
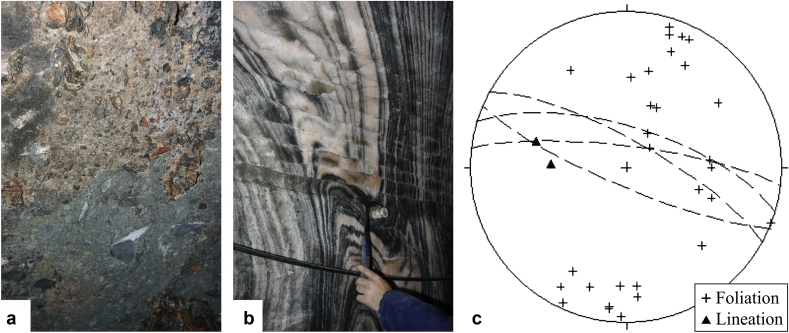
a – Protocataclastic Haselgebirge fabric with asymmetric and symmetric fringes of linear white salt behind clasts (Hörnerwerk, Kaiserin Elisabeth level). b – Folded rocksalt with an upright axial surface. c – Combined foliation data (poles to plane, black crosses, 30 data), axial plane foliation (great circles, 4 data), which indicates about NNE–SSW shortening and lineation data (2 data, black triangles) of all measured rocksalt of Kaiserin Maria Theresia level and Kaiserin Elisabeth level of the Hallstatt salt mine.

In general, the Haselgebirge Fm. shows a protocataclastic fabric (Fig. 7a) with variable sized clasts of sulphate rocks and mudstone in a halite matrix. Asymmetric and symmetric fringes of linear white salt behind clasts are common (Fig. 7a). Rocksalt and sulphates are deformed as ductile materials and show a pronounced foliation, which is often folded (Fig. 7b with an upright axial surface). Fold axes and stretching lineation are subhorizontal or plunge to the W or WNW (Fig. 7c). In some cases, we measured an axial plane foliation S_2b_, which is subvertical and trends toward WNW. This indicates ca. NNE–SSW shortening.

### 4.2 Sulphate fabrics

**Fig. 8:**
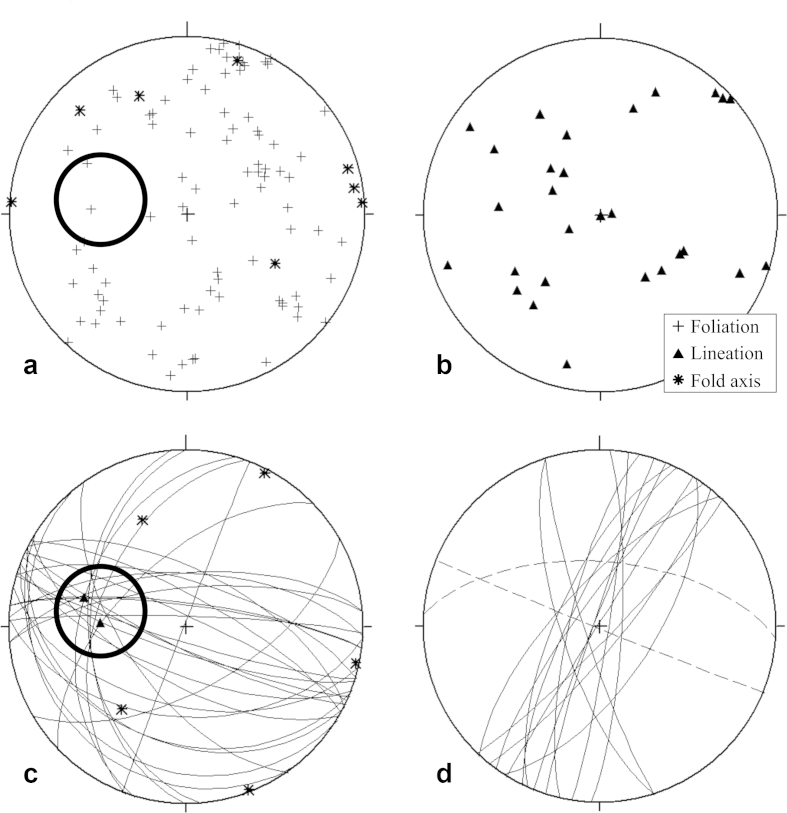
Structural data of the Hallstatt salt mine measured in Kaiserin Maria Theresia level and Kaiserin Elisabeth level: a – Combined foliation data of anhydrite and polyhalite rocks (pole to plane, black crosses, 89 data) and fold axis data of anhydrite and polyhalite rocks (black asterisks, 8 data); the circle (intersection of foliation great circles) marks the estimated fold axis at ∼280/20. b – Combined lineation data of all measured anhydrite and polyhalite rocks (28 data). c – Combined foliation data (great circles, 30 data), lineation data (2 data, black triangles) and fold axis data (black asterisks, 5 data) of all measured rocksalt outcrops; the circle (intersection of foliation great circles) marks the estimated fold axis at ∼280/20, which fits well to the lineation data. d – NNE- and N-trending, halite-filled extensional veins (solid lines) and E-trending, halite- and polyhalite-filled extensional veins (dashed lines) (great circles, 17 data).

**Fig. 9:**
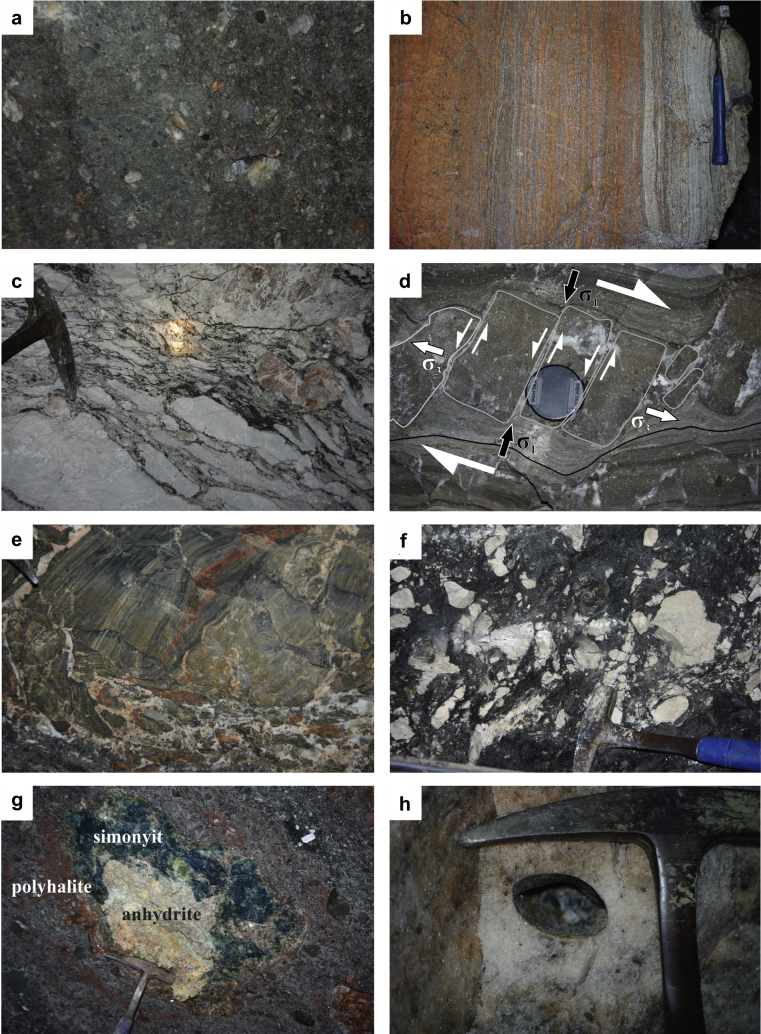
Underground photographs of the Hallstatt salt mine: a – Polymictic cataclasites with angular clasts (mainly claystone and subordinately red polyhalite), which are embedded in a matrix of rocksalt and mudstone (Sicherungswerk 3, Kaiserin Elisabeth level). b – Recrystallized polyhalite-anhydrite mylonite showing a distinct foliation (Sicherungswerk 3). c – Tectonic breccia within the “Central Inclusion” (Elisabeth-Hauptschachtricht (next to 450-plate), Kaiserin Elisabeth level). The nearly monomictic clasts of light-grey and red Hallstatt Limestone and anhydrite and polyhalite lenses were overprinted ductilely. d – A major dextral strike-slip fault, which dips steeply toward North and is formed by bookshelf mechanism, is outcropping within the dark-grey banded anhydrite of TWE-22 (Kaiserin Elisabeth level). The medium-grey banded anhydrite layers were disrupted and deformed ductilely, while the rheologically stronger dark-grey anhydrite layers were broken into fragments with small en echelon-veins filled with secondary white anhydrite. e – Recrystallized and foliated anhydrite, which is laterally broken to a monomictic breccia with a halite matrix, interbeds with a medium red polyhalite-boudin layer (Vernier-Kehr, Kaiserin Maria Theresia level). f – Tectonic breccia within the “Central Inclusion” (Elisabeth-Hauptschachtricht (next to 450-plate)), exposed at the contact to the lustrous slate. The breccia components are mainly composed of light-grey Hallstatt Limestone and subordinately rocksalt and anhydrite. g – About 0.5 × 0.5 cm large lens of dark-green simonyit, intergrown with light-grey anhydrite and halite and a medium-red polyhalite seam (Sicherungswerk 3). h – Elliptically deformed, subhorizontal borehole within rocksalt of Nördliche Hoffmann-Kehr (Kaiserin Maria Theresia level).

The foliation (Fig. 8a and Fig. 9b) and lineation (Fig. 8b) of anhydrite and polyhalite [K_2_Ca_2_Mg(SO_4_)_4_.2H_2_O] rocks at the Kaiserin Maria Theresia level and Kaiserin Elisabeth level are quite scattered, although the distribution of foliation surfaces argues for folding. The fold axis, which indicates a direction perpendicular to the greatest finite extension (Fig. 8a), is estimated at about 280/20 (intersection of foliation great circles) (Fig. 8a) and implies ca. NNE–SSW shortening. The distribution of stretching lineation of sulphates is highly variable and argues for a post-stretching rotation of the clast-like sulphate bodies.

### 4.3 Rocksalt fabrics

The measured rocksalt foliation and lineation data of Kaiserin Maria Theresia level and Kaiserin Elisabeth level (Fig. 8c) are also quite scattered. The ESE–WNW- trending axial plane foliation of rocksalt indicates about NNE–SSW shortening (Fig. 7c). The fold axis is estimated at about 280/20 (intersection of foliation great circles) (Fig. 8c). This is interpreted as a direction perpendicular to the greatest finite extension (see also Section 4.2. and Fig. 8a).

### 4.4 Extensional veins

**Fig. 10:**
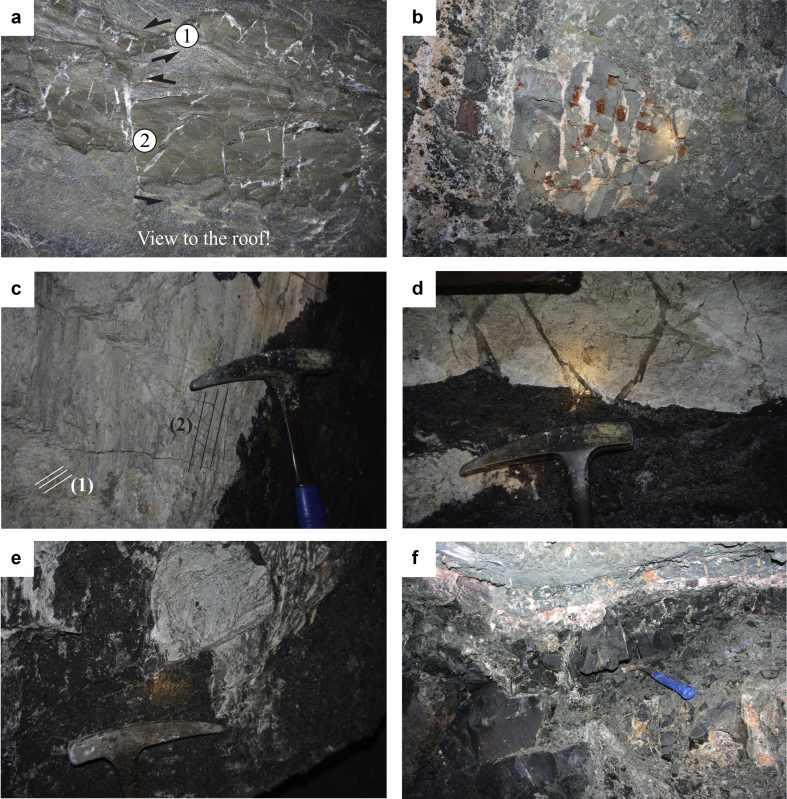
Underground photographs of the Hallstatt salt mine: a – A major apparent sinistral strike-slip fault is outcropping at the roof of TWE-22. Note the view to the roof, which indicates a dextral shear sense of the fault system. The medium-grey banded anhydrite layers show shear bands and were disrupted and deformed ductilely, while the mechanically stronger dark-grey anhydrite layers were fractured into fragments (see also Fig. 9d). The structures (1) and (2) are explained in the text. b – White halite-filled extensional veins within a mudstone clast (Hörnerwerk, Kaiserin Elisabeth level). c – Two generations of slickensides and striations at the contact of light-grey Hallstatt Limestone to the lustrous slate of the “Central Inclusion” (Elisabeth Hauptschachtricht (near 450-plate), Kaiserin Elisabeth level): The older movement (1) – a dextral strike-slip fault – shows a flat lineation, the younger reverse movement (2) a steep lineation. d – Light-grey Hallstatt Limestone with many halite-filled extensional veins (which indicate E–W extension) (upper part of the photograph) and lustrous slate (lower part of the photograph) within the “Central Inclusion” (Elisabeth-Hauptschachtricht). e – Hallstatt Limestone-mylonite within the “Central Inclusion“ (Elisabeth-Hauptschachtricht). f – Cataclastic brecciation of dark mudstone along the contact of a major grey mudstone layer. The matrix between the clasts is filled with halite.

Halite- and sulphate (anhydrite and polyhalite)- filled extensional veins show two main orientations (Fig. 8d and Fig. 10b): (1) NNE–SSW-trending, halite-filled extensional veins, indicating ESE–WNW extension. They might be interpreted as extensional veins (ac-joints) to the E–W-trending folds; (2) N–S-trending, halite- and anhydrite-filled transtensional veins, implying E–W extension. The veins are orientated at a high angle to the main orientation of the major dextral strike-slip fault (measured in banded anhydrite of Tiefenwerk Elisabeth 22 (=TWE-22 in the further text) of Kaiserin Elisabeth level) and they could also be interpreted as ac-joints to the E–W-trending folds. (3) WNW-trending halite-and polyhalite-filled, subvertical extensional veins occur subordinately, indicating NNE–SSW extension, which might be interpreted as bc-joints to the E–W-trending folds.

Folding of the rocksalt was mainly induced by NNE–SSW shortening as indicated by E–W-trending folds, E–W-trending veins (extensional veins to the E–W-trending folds) and vertical ESE–WNW-trending axial plane foliation.

### 4.5 Cataclasites

In the following we describe the various types of abundant cataclasites exposed at several levels of the Hallstatt rocksalt body. The most common rock types are polymictic cataclasites with a variety of angular clasts, which are embedded in a matrix of rocksalt and/or mudstone (Fig. 9a and Fig. 10f). The clasts often preserve internal ductile fabrics (Fig. 9b, e). Nearly monomictic clasts are also common along the margins of major inclusions, such as the Hallstatt Limestone (Fig. 9c, f). There, the components are mainly composed of light-grey Hallstatt Limestone and subordinately anhydrite- and polyhalite lenses. Some of these cataclasites are overprinted by subsequent ductile deformation, which led to elongation and pressure solution along rims at the contact to the shaly matrix (Fig. 9c). Other cataclasites with a similar clast composition do not show a secondary overprint (Fig. 9f).

This type of disruption process can be observed at several locations. The dark-grey banded anhydrite of TWE-22 (Fig. 9d and Fig. 10a) can be considered as an example for an initial stage of disruption along a steeply northward dipping dextral strike-slip shear zone over several tens of meters. The anhydrite layer was disrupted by bookshelf mechanism and the foliation of the enclosing foliated anhydrite is wrapping around the broken pieces (Fig. 9d and Fig. 10a). The medium-grey banded anhydrite layers were disrupted and deformed ductilely, while the rheologically stronger dark-grey anhydrite layers were broken into fragments. Between these about 8 × 8 cm, nearly rectangular, dark-grey anhydrite fragments small en echelon-veins were formed, which are filled with secondary white anhydrite. These transtensional zones are orientated about parallel to σ_1_ and represent the conjugate set to the overall dextral strike-slip-fault (Fig. 9d). The apparent sinistral strike-slip fault at the roof of TWE-22 (Fig. 10a) also indicates a dextral shear sense because of the view to the roof. Structure (1) is a shear band and structure (2) contains tilted boudins and implies a bookshelf mechanism. Both types of structures are formed by dextral shearing.

The foliated and recrystallized anhydrite breccia shown in Fig. 9e is a further example of a monomictic brecciated cataclasite. Continuous anhydrite layers interbedded with a red, recrystallized polyhalite-boudin layer are broken laterally and form a monomictic breccia within a halite matrix. This example shows the initiation of brecciation by reaching the yield strength of the anhydrite rock.

In some cases, metamorphic reactions occur between boudins, where angular anhydrite clasts might have reacted with halite and polyhalite to form aggregates of dark-green blödite [Na_2_Mg(SO_4_)_2_.4 H_2_O] (Fig. 9g) locally referred to as “Simonyit” (or Fe-astrakhanite). It serves as the index mineral for the red salt rock (“Rotsalzgebirge”) (Schauberger, 1986, Kirchner et al., 1981).

### 4.6 Brittle faults

The major inclusions as well as the borders of the Hallstatt evaporite body are often represented by black and graphite-bearing lustrous slate (“Glanzschiefer”). They indicate the movement of the rheologically weak rocksalt along the contact to the mechanically strong inclusions or country rocks. A light grey Hallstatt Limestone of the “Central Inclusion” (Elisabeth-Hauptschachtricht, Kaiserin Elisabeth level) (Fig. 10c–e) exposes at least two generations of slickensides and striations, with age relation that could be derived on-site at the subvertical E-trending contact to the lustrous slate. The older movement (1) - a dextral strike-slip fault - shows a subhorizontal lineation, the younger movement (2) was a reverse fault with a steep lineation (see Fig. 10c). The E-trending dextral strike-slip fault (1) (not shown), which probably formed by dextral shearing and brittle deformation, indicates NW–SE shortening and NE–SW extension. Furthermore, it is orientated about perpendicularly to the sinistral strike-slip fault measured within dark-grey anhydrite of TWE-22 (not shown), but it is not clear whether the dextral strike-slip fault or the sinistral strike-slip fault is older. The reverse fault (2) (not shown) indicates N–S shortening along a steep axis and is attributed to simple gravitative downward motion of the inclusion into the halite body.

Within the dark-grey anhydrite of TWE-22, a sinistral strike-slip fault indicating NE–SW shortening is orientated about perpendicularly to the dextral strike-slip fault within the Hallstatt Limestone of the “Central Inclusion”.

### 4.7 Recent strain

Because of the low strength of halite, the Hallstatt evaporite body is still subject to recent subvertical shortening and the strain rate of this process can be quantified by elliptically deformed subhorizontal boreholes (Fig. 9h).

The stretch *ε* was deduced as 1.27. The strain rate $\dot{\varepsilon }$ was calculated (as stretch/seconds between drilling and observation) in the following way:$\;\dot{\varepsilon }=\frac{1.27}{50\text{a}}=\frac{1.27}{1.825\times 10^{4}\text{d}}=\frac{1.27}{1.5768\times 10^{9}\text{s}}=8.038\;\times \;10^{-10}\;\left[{\text{s}}^{-1}\right]$

Thus, we quantified the strain rate $\dot{\varepsilon }$ as 8.04 × 10^−10^ [s^−1^]. This relatively high value is similar to strain rates (10^−10^ to 10^−9^ s^−1^) estimated by Leitner et al. (2011) from grain size analyses of halite from other salt mines within the NCA. The agreement between the values argues for a sub-recent (and possibly ongoing) formation of the steep planar halite microfabrics.

### 4.8 Succession of deformation phases

The presence of ductilely deformed sulphate rocks such as anhydrite and polyhalite, which were overprinted by ductile fabrics in rocksalt and cataclastically deformed by later deformation phases as well as the large-scale inferences, allow us to distinguish several deformation phases.

#### 4.8.1 Deformation phase D_1_

We consider polyhalite veins (Schorn et al., 2013a) preserved in tectonic lenses as the earliest recorded deformation. Similar veins in other salt mines were dated at between 235 and 230 Ma (Middle Triassic; Leitner et al., 2013a, Leitner et al., 2013b). No inference on orientation was made because of later rotation and preservation within meter- to decimeter-scaled tectonic lenses.

#### 4.8.2 Deformation phase D_2a, b_

We regard the steep mylonitic foliation S_2a_ and S_2b_ and stretching lineation L_2a_ and L_2b_ of sulphates (anhydrite and polyhalite rocks), which are potentially related to first order diapirism, as deformation phase D_2a, b_. Dating of mylonitic foliation in polyhalite rocks of the salt mine Altaussee yielded an age of ca. 118–105 Ma (Leitner et al., 2013b). Therefore, we consider an Early Cretaceous age of formation of this deformation fabric. The foliation and lineation might have formed during diapiric uprise of evaporites at elevated temperature conditions similar as proposed from analogue models of mushroom-shaped diapirs by Jackson and Talbot (1989), although the initial stages of foliation formation could be older. Ductile fabrics in large inclusions such as the Hallstatt Limestone are likely to have also formed during this tectonic event. On the map-scale (Fig. 4), the banded structure of rocksalt could have formed during this phase. However, in rocksalt, we are not able to distinguish between a foliation formed by diapiric processes (S_2a_) and subsequent strike-slip shearing (S_2b_).

#### 4.8.3 Deformation phase D_3_

During deformation phase D_3_, which indicates N–S shortening, the previously formed foliation S_2a, b_ in sulphate rocks was folded in outcrop-scale as open to tight folds with wavelengths of 0.2–1 m.

#### 4.8.4 Deformation phase D_4_

Deformation phase D_4_ represents the main phase of cataclastic deformation during a series of events. All four deformation phases D_1_–D_4_ have in common that cataclastic deformation occurred under low-temperature conditions and only halite always reacted, sulphate in part (D_2a, b_, D_3_) with ductile behavior.

#### 4.8.5 Deformation phase D_5_

Under deformation phase D_5_ we summarize the brittle structures especially along boundaries of inclusions. We particularly recognize brittle deformation with strike-slip deformation along ca. E-trending steep faults, which are part of a large-scale system (Fig. 2).

## 5 Discussion

In this section, the structural origin of the Hallstatt evaporite body is evaluated and a model for its deformation is proposed. The evaporite body is part of the NCA fold-thrust belt, which has developed through a succession of tectonic events from the late Early Cretaceous to the Miocene. The external maximum principal stresses changed their orientation in present-day coordinates from NE–SW over N–S to NW–SE back to N–S (Linzer et al., 1997, Peresson and Decker, 1997). Consequently, a multitude of events potentially shaped the Hallstatt evaporite body.

### 5.1 Early sulphate fabrics

The lenses of anhydrite and polyhalite rocks often preserve remnants of an earlier ductile fabric, which is not necessarily reflected by the rocksalt. As these ductile fabrics developed in the stability field of anhydrite and polyhalite, the temperature was confined by the lower stability of anhydrite (gypsum to bassanite [CaSO_4_.0.5H_2_O], ca. 100 °C, and bassanite to anhydrite conversion, ca. 140 °C; Brantut et al., 2011, Brantut et al., 2012) and by the upper stability of polyhalite (ca. 255 °C (255–343 °C, Wollmann et al., 2008) or 285 °C (Fischer et al., 1996); see Schorn et al., 2013a for discussion). The temperature limits of 140–255 (or up to 343) °C are well within the limits of what is known from studies using various geothermometers.

We suggest that these fabrics formed at elevated heat flow (Rantitsch and Russegger, 2005) during late Early Cretaceous tectonic events (deformation phase D_2a, b_) as the nappe stack of the NCA was formed and the Haselgebirge Fm. acted as a major décollement horizon (e.g., Linzer et al., 2002 and references therein).

### 5.2 Structure of the Hallstatt evaporite body

Referring to the works of Habermüller, 2005, Arnberger, 2006 and Schmid (2009) the Haselgebirge Fm. in the Hallstatt area is restricted to a major E- to ESE-trending dextral strike-slip fault zone at the northern boundary between the Hallstatt nappe and the Dachstein nappe. The ESE–WNW-striking salt body of Hallstatt is interpreted as a dextral strike-slip shear zone as indicated by a steeply oriented axial plane foliation and flat lying E–W-striking fold axes parallel to the mylonitic stretching lineation, which were also observed in deformation structures of the covering rocks. The dextral shear sense was also derived from porphyroclasts and 3D modeling of the salt body and the overlying units (Arnberger, 2006, Schmid, 2009). The dextral shear zone might be kinematically related to the WNW-directed thrusting (main transport direction in the Hallstatt area) as indicated by ESE-dipping thrust planes and associated ESE–WNW-trending striations on slickensides. This suggests that the evaporites acted as a detachment horizon for W-directed thrusting before their propagation into the strike-slip fault (Habermüller, 2005, Arnberger, 2006). Using a balanced cross-section through the northern part of the Hallstatt nappe a minimum shortening of 32.5% (Habermüller, 2005; 12% for the horse structure) or 42.1% (Arnberger, 2006) was estimated.

The root of the Hallstatt evaporite is not known and it is deeper than the level of Lake Hallstatt. As the geological cross-sections testify, the body is smaller at depth and widens upwards (Figs. 3 and 5). All mining maps and sections show a widening of the upper part and an elongation of the Haselgebirge Fm. towards the West. The widest part is at the eastern termination, where the Hallstatt body trends ca. NW–SE and where evidence of dextral shear is found. Internal structures are dominated by steep folds implying ca. NNE–SSW shortening.

As already mentioned in Section 3 the northern margin of the Hallstatt salt body dips steeply S, whereas the subvertical W to WNW-trending southern margin (Rossalm fault) (Figs. 2 and 3) includes both features of a reverse fault with a northern block up-kinematics and a strike-slip fault. This feature could be attributed to the superposition of two distinct events. At Kaiserin Maria Theresia level (Fig. 4) a nearly rectangular WNW-trending body in the East with thick lithologies indicating ca. NNE-SSW shortening can be distinguished from a small, heavily stretched and thinned W-trending “tail”, which was formed by sinistral shearing. Therefore, we conclude in reference to analogue models (see i.a. Jackson and Talbot, 1989, Hudec and Jackson, 2007 and references therein), that the eastern, less shortened part could be best explained as a compressive teardrop-like diapir with double-vergent thrusts (see Fig. 3, cross-section B), whereas the more sheared western part was deformed by subsequent processes.

Furthermore, we conclude that the large W-trending zone along the northern margin represents a transpressive ductile shear zone, which overprinted the diapir-like structure. We also note that besides the overall diapir-like structure of the Hallstatt body, evidence for structures, which resulted from diapiric rise, is weak except for subvertical striations and the foliation and lithologies parallel to the boundary of the evaporite body.

**Fig. 11:**
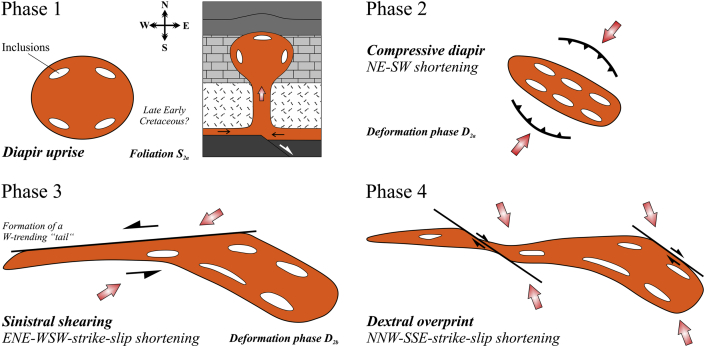
Model of the tectonic evolution of the Hallstatt evaporite body. Phases 1–4 are shown in map view, and Phase 1 also as vertical section. For explanation see text.

Summarizing the most important information, we recognized four different phases of deformation, which affected the Hallstatt evaporite body. As indicated by its mushroom diapir-like shape (see i.a. Jackson and Talbot, 1989, Hudec and Jackson, 2007 and references therein), we assume that the salt body of Hallstatt was formed by the uprise of rocksalt (Phase 1 in Fig. 11). We cannot evaluate the overall significance of the ductile sulphate fabrics (e.g. foliation S_2a_), which are preserved in meter- to ten meter-scaled lenses, although we suppose that these fabrics were formed during late Early Cretaceous. Furthermore, ductile fabrics in limestone might indicate a high-temperature ductile deformation during an early phase of Alpine deformation (see i.a. Fig. 10e). In a later phase, the evaporite body underwent ca. NE–SW shortening and a compressive teardrop-like diapir was formed (Phase 2 in Fig. 11, corresponding to deformation stage D_2a_). The evaporite body subsequently underwent sinistral shearing along a W-trending shear zone (see Phase 3 in Fig. 11, corresponding to deformation phase D_2b_), which was induced by ENE–WSW-strike-slip shortening. During this phase the westward extending “tail“ in the western part of the Hallstatt salt mine (see also Fig. 4) was formed. Finally, the “tail“ and the main body were affected by dextral displacement along NW-trending faults (Phase 4 in Fig. 11), which implies NW–SE-strike-slip shortening.

### 5.3 Significance and timing of deformation events

According to Arnberger (2006) and Schmid (2009) the youngest structural elements of the Haselgebirge mélange are veins, which mainly opened in E–W-direction and are formed by N–S shortening. The stretching lineation as well as the elongated halite fibres in fringes around clasts and N–S to NNE–SSW-trending subvertical extensional veins indicate a ca. E–W-orientation of the maximum instantaneous extension (see also Arnberger, 2006, Schmid, 2009).

### 5.4 Origin of cataclasites

Where the rocksalt content of the Haselgebirge Fm. is low, it represents a tectonic breccia or a cataclasite or protocataclasite (see also Leitner et al., 2011, Schorn and Neubauer, 2011, Schorn et al., 2013b). The clasts are rheologically strong rocks, which are either (1) part of the evaporite succession such as anhydrite and polyhalite rocks or (2) country rocks, which were incorporated during various phases of tectonic emplacement of the Hallstatt evaporite body. Based on subsurface observations, we argue that cataclasis was active during several deformation phases. However, all observations indicate that cataclasis postdates the ductile fabrics of sulphates (e.g., deformation phase D_2a, b_). In some cases, ductile deformation is observed in limestone inclusions (Fig. 9c), indicating still elevated temperatures.

The reason for cataclasis could be either high differential stresses or hydrofracturing due to the increase of fluid pressure. As high differential stresses are unlikely within a halite-dominated body, cataclasis was more likely formed by high fluid pressure. Elevated fluid pressure could be either caused by (1) the smectite-to-illite transformation (ca. 120–160 °C) of abundant mudstone, (2) gypsum to anhydrite dehydration (100–140 °C) or (3) invasion of water, e.g. by dehydration reactions in marly or shaly country rocks. We suggest that all three reasons could have played a significant role during specific phases of the tectonic evolution of the Hallstatt evaporite body.

### 5.5 Recent to present strain

The coincidence of our calculated strain rate and those estimated by Leitner et al. (2011) from the grain size distribution of halite strongly argues for a sub-recent formation of the halite microfabrics.

Recent deformation of the Hallstatt salt mine also primarily shows shortening in N–S-direction (personal comment Unterberger, 2003; quoted from Arnberger, 2006, Schmid, 2009). Accordingly, rail tracks in N–S-directed tunnels in the Hallstatt salt mine show severe bending of a wavelength of several tens of meters and an amplitude of ca. 8–10 cm, while no damages were observed in E–W-directed galleries.

On the other hand, subvertical strain as observed by elliptically deformed subhorizontal boreholes is also common (Fig. 9h). Although the GPS network is widely spaced, Caporali et al. (2013 and references therein) report a ca. northward motion of the study area in respect to stable Eurasia. This is in line with bending observed in N–S rail tunnels. In contrast, our data from the distorted borehole shows subvertical shortening. This could be explained by deformation through regional tectonics and/or differential loading by the overburden, particularly of the gravity-driven sinking of high-density anhydrite blocks (*ρ* = 2900 kg/m^3^) or other inclusions into the underlying halite-clay-rich Haselgebirge with a lower density (for rocksalt: *ρ* = 2200 kg/m^3^). Similar effects were proposed by Koyi (2001 and references therein) and Burchardt et al. (2012 and references therein). Subvertical shortening may have also contributed to N–S shortening by bending. However, more systematic and detailed future work in Hallstatt and other salt mines is needed to resolve details of these processes.

## 6 Conclusions

(1) According to results of analogue modeling reported in the literature, we interpret the external shape of the Hallstatt evaporite body as the effect of a compressive teardrop-like diapir.
(2) The external shape and internal structures are the result of superposition of diapiric uprise later transposed by ca. N–S to NNE–SSW shortening and subsequent lateral shear.
(3) The main rock types of the salt body include protocataclasites and rocksalt shear zones. Early fabrics of ductile shear zones are preserved in the anhydrite and polyhalite rocks, which likely formed due to late Early Cretaceous deformation.
(4) A recent strain rate $\dot{\varepsilon }$ of 8 × 10^−10^ [s^−1^] is deduced from deformed subhorizontal boreholes.

## References

[bib1] Arnberger K. (2006). Detachment Folding above the Permian Haselgebirge: Palinspastic Reconstruction of Alpine West-directed Thrusting (Plassen, Hallstatt, Upper Austria).

[bib2] Brantut N., Han R., Shimamoto T., Findling N., Schubnel A. (2011). Fast slip with inhibited temperature rise due to mineral dehydration: evidence from experiments on gypsum. Geology.

[bib3] Brantut N., Schubnel A., David E.C., Héripré E., Guéguen Y., Dimanov A. (2012). Dehydration-induced damage and deformation in gypsum and implications for subduction zone processes. J. Geophys. Res. Solid Earth.

[bib4] Butler R.W.H., Paton D.A. (2010). Evaluating lateral compaction in deepwater fold and thrust belts: how much are we missing from “nature’s sandbox”?. GSA Today.

[bib5] Burchardt S., Koyi H., Schmeling H., Fuchs L. (2012). Sinking of anhydrite blocks within a Newtonian salt diapir: modelling the influence of block aspect ratio and salt stratification. Geophys. J. Int..

[bib6] Caporali A., Neubauer F., Stangl G., Zuliani D. (2013). Modeling surface GPS velocities in the Southern and Eastern Alps by finite dislocations at crustal depths. Tectonophysics.

[bib7] Dallmeyer R.D., Neubauer F., Fritz H., Siegesmund S., Fügenschuh B., Froitzheim N. (2008). The Meliata suture in the Carpathians: regional significance and implications for the evolution of high-pressure wedges within collisional orogens. Tectonic Aspects of the Alpine-Dinaride–Carpathian System.

[bib8] Faupl P., Wagreich M. (2000). Late Jurassic to Eocene paleogeography and geodynamic evolution of the Eastern Alps. Mittl. Österreichischen Geol. Ges..

[bib9] Fischer S., Voigt W., Köhnke K. (1996). The thermal decomposition of polyhalite 2SO_4_.MgSO_4_.2CaSO_4_.2H_2_O. Cryst. Res. Technol..

[bib10] Gawlick H.-J., Schlagintweit F. (2006). Berriasian drowning of the Plassen carbonate platform at the type-locality and its bearing on the early Eoalpine orogenic dynamics in the Northern Calcareous Alps (Austria). Int. J. Earth Sci..

[bib11] Habermüller M. (2005). West-directed Thrusting in the Dachstein Nappe: Quantification of the Eo-Alpine Deformation Around the Echerntal Valley (Hallstatt, Austria).

[bib12] Hudec M.R., Jackson M.P.A. (2007). Terra Infirma: understanding salt tectonics. Earth Sci. Rev..

[bib13] Jackson M.P.A., Talbot C. (1989). Anatomy of mushroom-shaped diapirs. J. Struct. Geol..

[bib14] Kern A., Kowarik K., Rausch A.W., Reschreiter H. (2008). Salz – Reich 7000 Jahre Hallstatt. Veröffentlichungen der Prähistorischen Abteilung (VPA) 2.

[bib15] Kirchner E., Meixner H., Höll R., Mostler H., Schauberger O., Seemann R. (1981). Exkursionen zu den Lagerstätten und Mineralvorkommen innerhalb der Grauwackenzone, des Tauernfensters (Schieferhülle) und der nördlichen Kalkalpenbasis im zentralen Teil Österreichs. Fortschritte Mineral. 59 Beih..

[bib16] Klaus W. (1965). Zur Einstufung alpiner Salztone mittels Sporen. Verhandlungen der Geologischen Bundesanstalt Sonderheft G.

[bib17] Klaus W. (1987). Einführung in die Paläobotanik. Fossile Pflanzenwelt und Rohstoffbildung.

[bib18] Kober L. (1955). Bau und Entstehung der Alpen.

[bib19] Koyi H.A. (2001). Modeling the influence of sinking anhydrite blocks on salt diapirs targeted for hazardous waste disposal. Geology.

[bib20] Krystyn L. (2008). Exkursion 1. The Hallstatt pelagics – Norian and Rhaetian Fossillagerstaetten of Hallstatt. Berichte Geol. Bundesanst..

[bib21] Lein R., Flügel H.W., Faupl P. (1987). Evolution of the Northern Calcareous Alps during Triassic times. Geodynamics of the Eastern Alps.

[bib22] Leitner C., Neubauer F. (2011). Tectonic significance of structures within the salt deposits Altaussee and Berchtesgaden–Bad Dürrnberg, Northern Calcareous Alps. Aust. J. Earth Sci..

[bib23] Leitner C., Neubauer F., Urai J.L., Schoenherr J. (2011). Structure and evolution of a rocksalt-mudrock-tectonite: the haselgebirge in the Northern Calcareous Alps. J. Struct. Geol..

[bib24] Leitner C., Neubauer F., Marschallinger R., Genser J., Bernroider M. (2013). Origin of deformed halite hopper crystals, pseudomorphic anhydrite cubes and polyhalite in Alpine evaporites (Austria, Germany). Int. J. Earth Sci. (Geologische Rundschau).

[bib25] Leitner C., Neubauer F., Borojević-Šoštarić S., Genser J., Rantitsch G., Jourdan F., Mark D.F., Verati C. (2013). ^40^Ar/^39^Ar ages of crystallization and recrystallization of rock-forming polyhalite in Alpine rocksalt deposits. Advances in ^40^Ar/^39^Ar Dating from Archaeology to Planetary Sciences.

[bib26] Linzer H.-G., Moser F., Nemes F., Ratschbacher L., Sperner B. (1997). Build-up and dismembering of the eastern Northern Calcareous Alps. Tectonophysics.

[bib27] Linzer H.-G., Decker K., Peresson H., DelĺMour R., Frisch W. (2002). Balancing lateral orogenic float of the Eastern Alps. Tectonophysics.

[bib28] Mandl G.W. (1982). Jurassische Gleittektonik im Bereich der Hallstätter Zone zwischen Bad Ischl und Bad Aussee (Salzkammergut Österreich). Mitteilungen der Gesellschaft der Geologie- und Bergbaustudenten in Österreich.

[bib29] Mandl G.W. (1998). Geologische Karte der Dachsteinregion 1:50.000. Archiv für Lagerstättenforschung der Geologischen Bundesanstalt 21 (2001), Beilage 1.

[bib30] Mandl G.W. (2000). The Alpine sector of the Tethyan shelf – example of Triassic to Jurassic sedimentation and deformation from the Northern Calcareous. Mittl. Österreichischen Geol. Ges..

[bib31] Mandl G.W., van Husen D., Lobitzer H. (2012). Erläuterungen zu Blatt 96 Bad Ischl, Geologische Karte der Republik Österreich 1:50 000.

[bib32] Missoni S., Gawlick H.-J. (2011). Evidence for Jurassic subduction from the Northern Calcareous Alps (Berchtesgaden; Austoalpine, Germany). Int. J. Earth Sci..

[bib33] Neubauer F., Genser J., Handler R. (2000). The Eastern Alps: result of a two-stage collision process. Mittl. d. Österreichischen Geol. Ges..

[bib34] Ortner H., Reiter F., Acs P. (2002). Easy handling of tectonic data: the programs TectonicVB for Mac and TectonicsFP for Windows(TM). Comput. Geosci..

[bib35] Peresson H., Decker K. (1997). The Tertiary dynamics of the northern Eastern Alps (Austria): changing Paleostress in a collisional plate boundary. Tectonophysics.

[bib36] Pichler H. (1963). Geologische Untersuchungen im Gebiet zwischen Rossfeld und Markt Schellenberg im Berchtesgadener Land. Beih. Zum Geol. Jahrb..

[bib37] Plöchinger B. (1982). Geologische Karte der Republik Österreich, Blatt 95 Sankt Wolfgang im Salzkammergut.

[bib38] Plöchinger B. (1984). Zum Nachweis jurassischer-kretazischer Eingleitungen von Hallstätter Gesteinsmassen beiderseits des Salzach-Quertales (Salzburg). Geol. Rundsch..

[bib39] Rantitsch G., Russegger B. (2005). Organic maturation within the Central Northern Calcareous Alps (Eastern Alps). Aust. J. Earth Sci..

[bib40] Ratschbacher L. (1986). Kinematics of Austro-Alpine cover nappes: changing translation path due to transpression. Tectonophysics.

[bib41] Schäffer G. (1982). Geologische Karte der Republik Österreich 1:50.000, Blatt 96 Bad Ischl.

[bib42] Schauberger O. (1949). Die stratigraphische Aufgliederung des alpinen Haselgebirges. Berg- Hüttenmännische Monatsh..

[bib43] Schauberger O. (1955). Zur Genese des alpinen Haselgebirges. Z. Dtsch. Geol. Ges..

[bib44] Schauberger O. (1986). Bau und Bildung der Salzlagerstätten des ostalpinen Salinars. Arch. für Lagerstättenforsch. Geol. Bundesanst..

[bib45] Schauberger O., Medwenitsch W. (1951). Der Hallstätter Erbstollen (Salzberg Hallstatt). Verhandlungen der Geologischen Bundesanstalt.

[bib46] Scheidleder A., Boroviczeny F., Graf W., Hofmann T., Mandl G.W., Schubert G., Stichler W., Trimborn P., Kralik M. (2001). Pilotprojekt “Karstwasser Dachstein”. Band 2: Karsthydrologie und Kontaminationsrisiko von Quellen. Archiv für Lagerstättenforschung der geologischen Bundesanstalt.

[bib47] Schmid N. (2009). Detachment Folding and Salt Tectonics in the Permian Haselgebirge (Hallstatt, Northern Calcareous Alps).

[bib48] Schorn A., Neubauer F. (2011). Emplacement of an evaporitic mélange nappe in central Northern Calcareous Alps: evidence from the Moosegg klippe (Austria). Aust. J. Earth Sci..

[bib49] Schorn A., Neubauer F., Bernroider M. (2013). Polyhalite microfabrics in an Alpine evaporite mélange: Hallstatt, Eastern Alps. J. Struct. Geol..

[bib50] Schorn A., Neubauer F., Genser J., Bernroider M. (2013). The Haselgebirge evaporite mélange in central Northern Calcareous Alps (Austria): Part of the Permian to Lower Triassic rift of the Meliata ocean?. Tectonophysics.

[bib51] Schweigl J., Neubauer F. (1997). Structural development of the central Northern Calcareous Alps: significance for the Jurassic to Tertiary geodynamics in the Alps. Eclogae Geol. Helvetica.

[bib52] Spötl C. (1987). Eine klastisch-evaporitische Oberperm-Entwicklung im Hallstätter Salzberg (Salzkammergut, Österreich). Mittl. Österreichischen Geol. Ges..

[bib53] Spötl C. (1988). Schwefelisotopendatierungen und fazielle Entwicklung permosythischer Anhydrite in den Salzbergbauen von Dürrnberg/Hallein und Hallstatt (Österreich). Mitteilungen der Gesellschaft der Geologie- und Bergbaustudenten in Österreich.

[bib54] Spötl C. (1988). Sedimentologisch-fazielle Analyse tektonisierter Evaporitserien – eine Fallstudie am Beispiel des Alpinen Haselgebirges (Permoskyth, Nördliche Kalkalpen). Geologisch-Paläontologische Mitteilungen Innsbruck.

[bib55] Spötl C. (1989). The Alpine Haselgebirge Formation, Northern Calcareous Alps (Austria). Permo-Scythian evaporites in an alpine thrust system. Sediment. Geol..

[bib56] Spötl C., Pak E. (1996). A strontium and sulfur isotopic study of Permo-Triassic evaporites in the Northern Calcareous Alps, Austria. Chem. Geol..

[bib57] Spötl C., Hasenhüttl C. (1998). Thermal history of an evaporitic mélange in the northern Calcareous Alps (Austria): a reconnaissance illite ‘crystallinity’ and reflectance study. Geol. Rundsch..

[bib58] Suzuki H., Gawlick H.-J. (2009). Jurassic Radiolarians from Cherty Limestones below the Hallstatt Salt Mine (Northern Calcareous Alps, Austria). Neues Jahrbuch für Geologie und Paläontologie Abhandlungen.

[bib59] Tollmann A. (1985). Geologie von Österreich. Außerzentralalpiner Anteil.

[bib60] Wagreich M., Decker K. (2001). Sedimentary tectonics and subsidence modeling of the type Upper Cretaceous Gosau basin (Northern Calcareous Alps, Austria). Int. J. Earth Sci. (Geologische Rundschau).

[bib61] Warren J.K. (2006). Evaporites. Sediments, Resources and Hydrocarbons.

[bib62] Wollmann G., Freyer D., Voigt W. (2008). Polyhalite and its analogous triple salts. Monatsh. für Chem..

[bib63] Zirkl E.J. (1957). Der Melaphyr von Hallstatt. Jahrbuch der Geologischen Bundesanstalt.

